# Inductively guided circuits for ultracold dressed atoms

**DOI:** 10.1038/ncomms6289

**Published:** 2014-10-28

**Authors:** German A. Sinuco-León, Kathryn A. Burrows, Aidan S. Arnold, Barry M. Garraway

**Affiliations:** 1 Department of Physics and Astronomy, University of Sussex, Falmer, Brighton BN1 9QH, UK; 2 Department of Physics, SUPA, University of Strathclyde, Glasgow G4 0NG, UK

## Abstract

Recent progress in optics, atomic physics and material science has paved the way to study quantum effects in ultracold atomic alkali gases confined to non-trivial geometries. Multiply connected traps for cold atoms can be prepared by combining inhomogeneous distributions of DC and radio-frequency electromagnetic fields with optical fields that require complex systems for frequency control and stabilization. Here we propose a flexible and robust scheme that creates closed quasi-one-dimensional guides for ultracold atoms through the ‘dressing’ of hyperfine sublevels of the atomic ground state, where the dressing field is spatially modulated by inductive effects over a micro-engineered conducting loop. Remarkably, for commonly used atomic species (for example, ^7^Li and ^87^Rb), the guide operation relies entirely on controlling static and low-frequency fields in the regimes of radio-frequency and microwave frequencies. This novel trapping scheme can be implemented with current technology for micro-fabrication and electronic control.

## Abstract (web summary)

Ultracold atomic gases show interesting quantum effects but the traps needed to study them are complex and often unwieldy. This study proposes a flexible and robust trapping scheme based on a spatially modulated atomic dressing field, created from an inductive loop, that traps atoms in one-dimensional guides.

## Graphical abstract

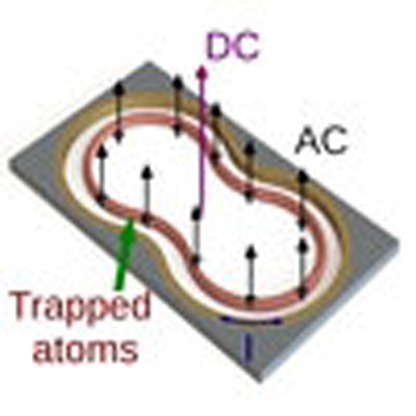

Ultracold atomic gases of alkali atoms are suitable for exploring fundamental questions in physics and developing quantum technologies. Such a double utility stems from the possibility of varying the interatomic interaction and potential landscape, through electromagnetic fields that can be precisely produced. Advances in this area have lead to impressive experimental demonstrations of macroscopic quantum phenomena such as matter-wave interferometry^1^ and persistent matter flux^2^,^3^. This is possible because appropriately tuned radiation addresses the atomic level structure and affects the dynamics of both internal as well as motional degrees of freedom^4^. This approach has been exploited to create complex miniaturized potentials for the atomic motion, utilizing laser radiation that addresses optical dipole transitions. Thus, the features of the resulting potential landscape vary with a length-scale limited by optical diffractive effects, being of the same order of magnitude as the laser wavelength and corresponding to a few hundred nanometres for alkali atoms.

In contrast, atom chips can create microscopic trapping structures utilizing long wavelength radiation^5^. In this case, the atomic potential landscape is tailored on a micron scale using electromagnetic fields radiating from micron-sized conductors. Developments in this area have made it possible to address atomic energy levels separated by low-frequency photons (radio-frequency and microwave) where decoherence effects are substantially reduced in comparison with optical transitions. These ideas were central for achieving radio-frequency-assisted coherent splitting of Bose-Einstein condensates for the first time in ref. 1, and, more recently, for beating the standard quantum limit with a scanning probe atom interferometer^6^. Micron and sub-micron control over atomic gases is at the heart of promising technological applications in metrology^6^,^7^, quantum information technology^8^ and quantum simulation^9^,^10^, and atom chips are platforms with great potential for experimental realization of many of such proposals.

Microscopic ring traps (and toroidal traps) are of particular interest because of the possibility they offer to study physical phenomena in a non-trivial geometry with true periodic boundary conditions and to create atomic analogues of solid state electronic devices (for example refs 7, 11). Trapping of cold gases in such geometries has been demonstrated with a variety of experimental techniques, requiring control over optical fields^2^,^3^,^12^,^13^,^14^ or distributions of magnetic fields^15^,^16^,^17^,^18^. There are several proposals for creating ring traps that rely solely on the field produced by DC current carrying conductors, suitable to be implemented with atom-chip technology (for example, refs 17, 19), but having the downside that feed wires can break desirable symmetries. Such an effect can be mitigated by employing low-frequency inductive coupling^20^, where the atoms are confined by time-averaged magnetic potentials. This idea has been experimentally demonstrated in millimetre-sized ring traps^18^ and proposed to produce microscopic ring traps based on generalizing the radio-frequency dressing approach^21^ to an inductive system^22^.

In this work, we show that highly configurable quasi-one-dimensional microscopic guides for ultra-cold alkali atoms result from the response of an inductive loop to AC magnetic fields tuned near the atomic ground state *hyperfine* splitting. We find various advantages of this approach over optical schemes and dressing of Zeeman split levels (that is, coupling states within the same hyperfine manifold)^20^,^22^. For example, the present proposal does not require sophisticated optical control and it is free from potential symmetry breaking current carrying wires in the vicinity of the trapping volume^19^,^23^,^24^,^25^,^26^. In addition, our system can be designed to create multiply connected atomic circuits, for example, arrays of connected ring traps, having in mind applications that benefit from matter-wave interferometry as in ref. 7. Importantly, in this work we also demonstrate the experimental feasibility of our scheme using current atom-chip technology^5^.

## Results

### Device concept and trapping mechanism

**Figure 1:**
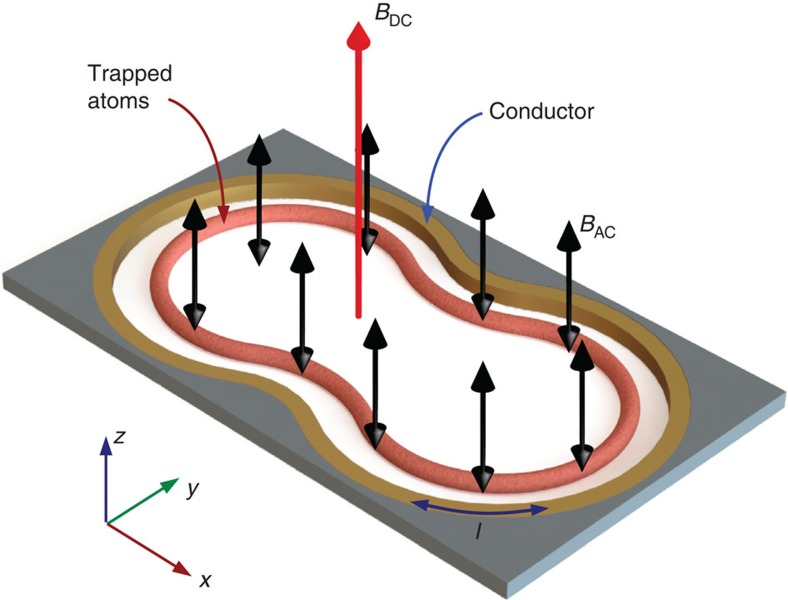
Elements of an inductively coupled guide for cold atoms. Atom-chip configuration creating an inductively coupled guide for ultracold atoms. It shows the magnetic field configuration (arrows), a closed conductor (yellow) and the generated trapping region (red).

**Figure 2:**
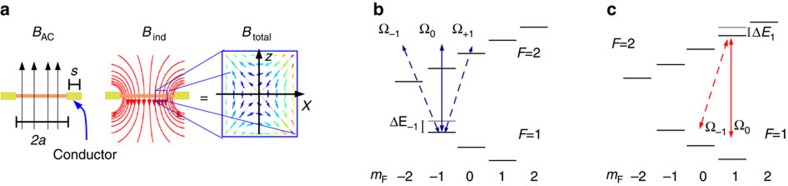
Field configuration and level couplings. (**a**) Side view of the AC magnetic field distribution in the neighbourhood of the conductor: the uniform external field (left) combines with the induced field (centre) and produces a total field with a quadrupole-like distribution close to the conductor (right). (**b**,**c**) Ground state energy level structure of ^87^Rb and magnetic couplings of two sub-levels (**b**) |*F*=1, *m*_*F*_=−1› (**c**) |*F*=2, *m*_*F*_=1›. Arrows indicate magnetic dipole couplings between pairs of hyperfine sub-levels, corresponding to linear (solid lines) and circular (dashed lines) polarizations of the magnetic field.

A sketch of the physical set-up is shown in Fig. 1. It comprises a micro-engineered conducting loop (metallic or superconducting), a static magnetic field 
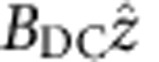
 (single-headed arrow), and a homogeneous AC magnetic field 
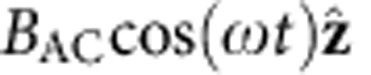
 (double-headed arrows). Both fields are transverse to the plane defined by the loop (*x*−*y* in Fig. 1). In response to the electro-motive force induced by the time variation of the magnetic flux across the area enclosed by the loop, an electric current circulates within it. The induced current produces, in its turn, an inhomogeneous magnetic field of the form **B**_ind_(**r**) cos(*ωt*+*δ*), that modifies the total AC magnetic field. For sufficiently large frequencies that the inductive reactance of the loop dominates its Ohmic resistance, the external and induced fields are almost in anti-phase. Thus, the resulting field has an approximately quadrupole distribution, schematically shown in Fig. 2a, whose centre is located close to the conducting loop at the position where the amplitude of induced and external fields satisfy *B*_ind_=*B*_AC_ cos(*δ*) (ref. 20), where *δ*+*π* is the relative phase between external and induced fields.

By tuning the driving angular frequency *ω* near the atomic ground state hyperfine transition, the AC magnetic field couples hyperfine Zeeman split sub-levels as depicted in Fig. 2b,c. The induced energy shifts lead to state-dependent potential energy landscapes for the atomic centre-of-mass motion, which have been used for weakly trapping neutral alkali atoms^27^,^28^ and coherent manipulation of Bose-Einstein condensates^29^. The electric field associated with the oscillating magnetic field can be ignored, as the time-averaged quadratic Stark shift is proportional to the atomic DC polarizability of the ground state and thus independent of the quantum numbers *F* and *m*_F_ (ref. 30). The energy shifts are conveniently described in terms of the field components in spherical unit vectors 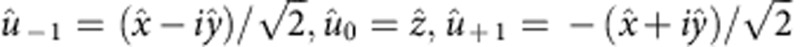, and corresponding Rabi frequencies 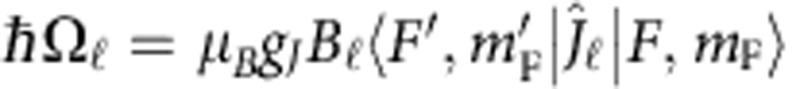 with *ℓ*=−1,0,1 and *g*_*J*_ the Landé factor of the electronic angular momentum *J*. After the rotating-wave approximation and utilizing second order perturbation theory, near the quadrupole centre the energy shifts are given by^30^


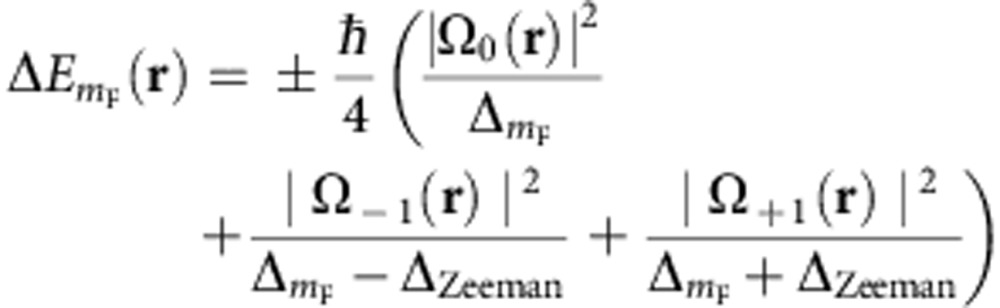


with *ℏ*Δ_Zeeman_=*μ*_B_*g*_F_*B*_DC_ and the detuning


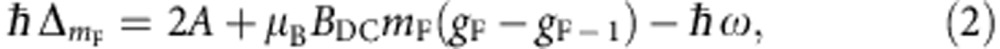


where the zero field hyperfine splitting of the ground state is 2*A*, and *g*_F_ the hyperfine Landé factor. The ± sign in equation (1) corresponds to the states of the hyperfine manifolds *F*=*I*±1/2. Dynamical control over the potential landscape can be exerted via the amplitude of the applied fields and the detuning. With respect to these parameters, trapping characteristics scale in a similar manner to those of optical dipole traps^14^. First, the trap depth, defined as the difference between the energy at the trap centre (**r**_0_:=(*x*_0_,0,0)) and at the geometrical centre of the conducting ring (**r**:=(0,0,0)), is proportional to the power of the applied AC field and the inverse of the detuning. Second, the trap frequency scales in proportion to the amplitude of the field and the inverse of the square root of the detuning. Notice also that as a consequence of the linear relation of induced current with both the amplitude *B*_AC_ and angular frequency *ω* of the applied field, the location of the trap centre (*x*_0_) is extremely robust to noise on both parameters. In addition, as shown below, for typical working parameters the ring trap is sufficiently far from the edge of the conducting ring that proximity effects can be neglected.

**Figure 3:**
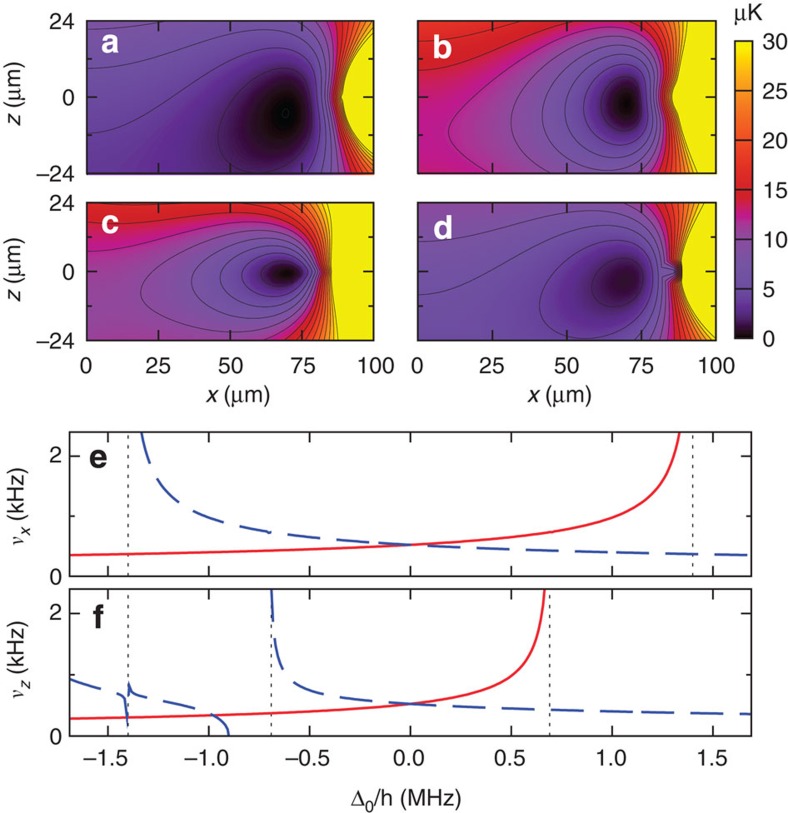
Trapping landscapes and frequencies. (**a**–**d**) Cross section of the trapping potential corresponding to Δ_0_/2*π*= −1.1 MHz (left column) and Δ_0_/2*π*=0.5 MHz (right column), for the states (**a**,**b**) |*F*=2, *m*_F_=1› and (**c**,**d**) |*F*=1, *m*_F_=−1›. Gravitational attraction is included. (**e**,**f**) Trap frequencies corresponding to states |*F*=2, *m*_F_=1› (solid) and |*F*=1, *m*_F_=−1› (dashed) of ^87^Rb, as a function of the AC detuning, with *B*_AC_=2 G, *B*_DC_=1 G, along the (**e**) *x* and (**f**) *z* directions. Vertical dotted lines in (**e**) and (**f**) indicate frequencies of resonant driving.

For illustrative purposes, we present calculations for the hyperfine level structure of ^87^Rb, denoted by |*F*, *m*_F_›, and shown in Fig. 2b,c. Nevertheless, our conclusions are straightforwardly extended to other atomic species with similar energy level structure. To give an explicit example of the potential landscape emerging from equation (1), we consider a circular loop of gold with radius *a*=100 μm and diameter *s*=10 μm, corresponding to approximate resistance *R* ≈0.26 Ω and inductance *L* ≈0.33 nH (ref. 31). In this case, the total field distribution produces a circular trapping region with typical landscapes as shown in Fig. 3a–d, for states |*F*=2, *m*_F_=1› and |*F*=1, *m*_F_=−1› of ^87^Rb, and applied fields of *B*_DC_=1 G and *B*_AC_=2 G. Notice that in this example the centre of the quadrupole distribution is located at *x*_0_ ≈70 μm, corresponding to a distance of ≈20 μm from the edge of the conducting ring.

The quadrupole AC field distribution produces harmonic confinement, as the linear dependence of the field amplitude with the distance to the quadrupole centre translates into a quadratic variation of the energy shift in equation (1). The tightness of the trap, quantified by the spatial curvature of 
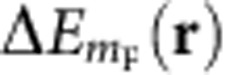
 along the 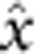 and 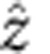 directions, is shown in Fig. 3e,f as a function of the detuning of the driving field (see equation (2)). According to equation (1), the trapping tightness increases arbitrarily by reducing the detuning with respect to pairs of transitions, resulting in the divergent behaviour in Fig. 3e,f (vertical dashed lines) at integer multiples of 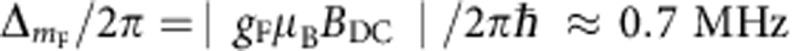 for *B*_DC_=1 G.

This trapping scheme provides confinement of two hyperfine states in overlapping regions, which can be useful for applications in quantum information processing and metrology^6^,^8^. In our example of Fig. 3e,f, detuning in the range Δ_0_/2*π*ε[−0.5, 0.5]MHz produces energy-shift landscapes for states |*F*=2, *m*_F_=1› and |*F*=1, *m*_F_=−1› with approximately equal curvatures for both states. These states experience exactly the same potential landscape for a driving field resonant to the hyperfine splitting, that is, with Δ_0_=0. Note that the static magnetic field makes this resonant driving blue (red) detuned with respect to coupling of states with *m*_F_=−1 (*m*_F_=1), as schematically shown by the solid arrows in Fig. 2b (Fig. 2c).

The detuning of the driving field also provides control over the shape of the trapping cross section, as seen in the potential landscapes in Fig. 3a–d. This is because the relative weights of the terms in equation (1) can be adjusted by changing the offset field and the driving frequency that determine 
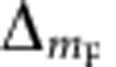
.

### Connected double loop trap

**Figure 4:**
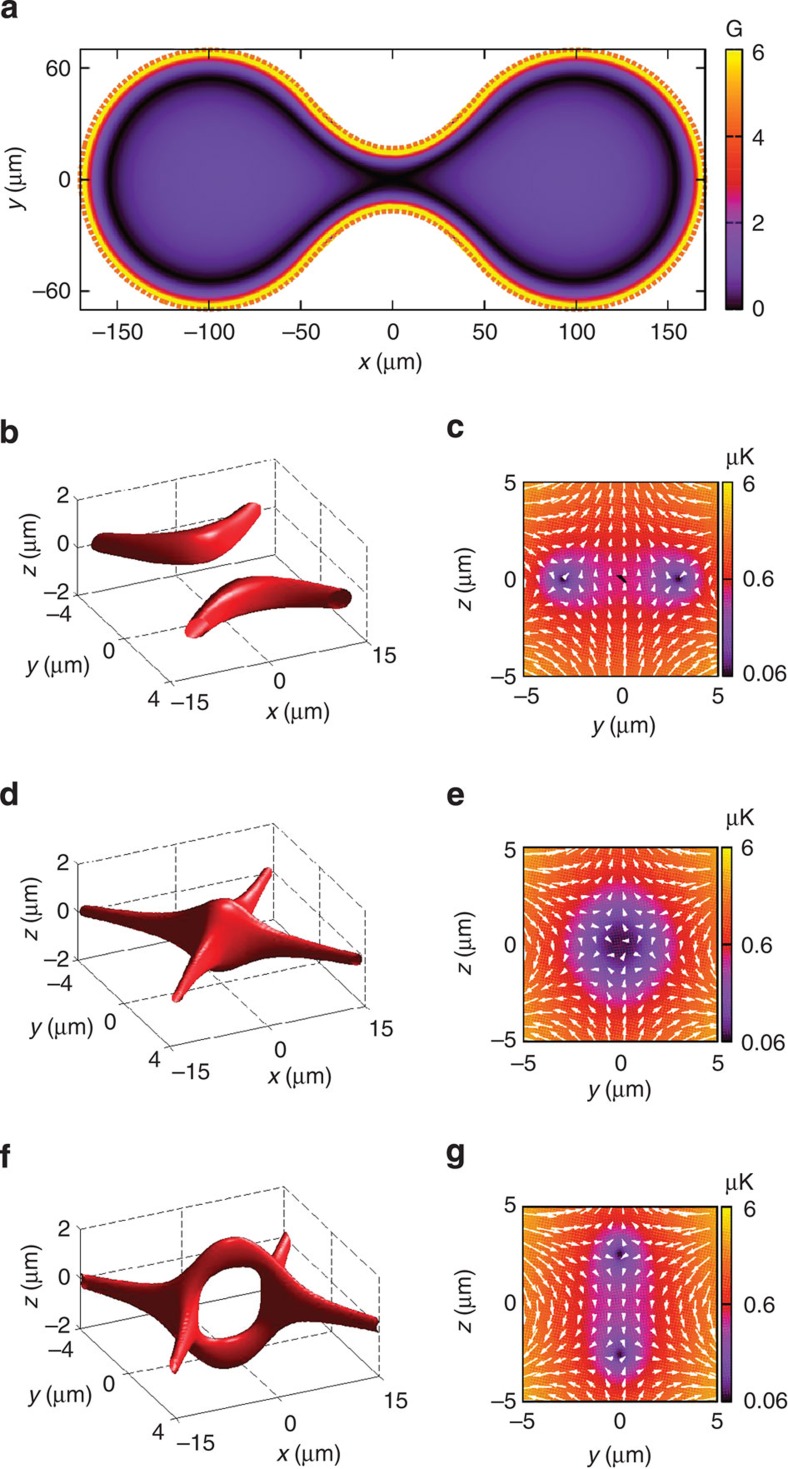
Connected loop geometry. A figure-of-eight guide for atoms in the state |*F*=2, *m*_*F*_ =1› of ^87^Rb, produced by a loop with a central symmetric constriction (orange dashed line in (**a**)). The conductor shape is defined by circles of radius 70 μm centred at *x*=±100 μm and a pair of parabolas that cuts the circle with matching first derivative. (**a**) Magnetic field landscape in the loop plane, *z*=0, for the applied fields *B*_DC_=1 G, *B*_AC_=2 G. (**b**–**g**) Left column: iso-energy surface at 0.5 μK corresponding to central gaps of (**b**) 35.2 μm, (**d**) 33.9 μm and (**f**) 32.9 μm. Right column: potential energy landscape and field distribution in the plane *x*=0, corresponding to surface plots on the left column. In (**b**–**g**) Δ_0_/2*π*=0.35 MHz and the colour bar is in logarithmic scale.

So far we have focused on the trapping produced by a circular conductor. However, our scheme offers the possibility of creating complex atomic guides shaped by the conducting loop. We illustrate this by considering a demanding case where we impose a severe ‘pinch’ in the shape of the conducting loop, as depicted in Fig. 4, creating a double loop with a variety of junction geometries. The field distribution corresponding to this case can be understood as follows: away from the pinch centre, the field distribution is similar to the quadrupole field in Fig. 1c, while in its neighbourhood the total field results from combining two quadrupole-like distributions associated with conducting segments at each side of the constriction. In particular, when the induced field balances the applied one at the centre of the pinch, the field distribution acquires a hexapolar character. The geometry of the resulting potential landscape is sensitive to the shape of the conductor, while its energy scale is determined by the amplitude and detuning of the applied fields. This is illustrated in Fig. 4b,g, where field distributions and energy landscapes have been obtained for three different constrictions with sizes differing by ≈1 μm, producing significantly different junction geometries. Consideration of this case can be straightforwardly applied to more complex geometries of the conductor, which can be used to create more involved atomic guides.

### Finite size effects

**Figure 5:**
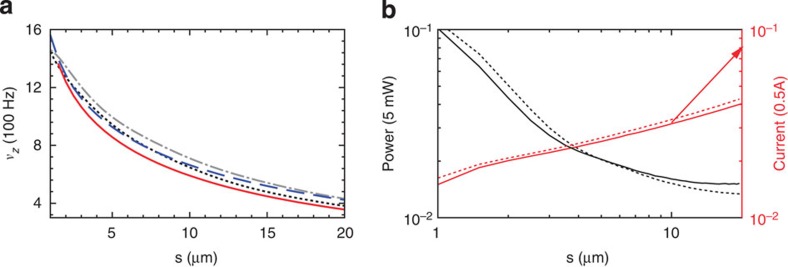
Finite size effects over the trap and the induced current. (**a**) Trap frequency (in 100 Hz) as a function of conductor thickness (*s*), corresponding to circular and square cross sections of superconducting Nb (solid and dashed lines) and gold (short-dashed and dot-dashed lines). (**b**) Peak values of power dissipated (in factors of 5 mW, black lines) and total current (in factors of 0.5A, red lines) in gold loops of circular (solid) and square cross sections (dashed). To obtain these results, we considered a circular loop of radius *a*=100 μm, applied fields *B*_AC_=2 G, *B*_DC_=1 G and detuning Δ_0_=0.

Modelling the loop as a single current filament is insufficient to describe the potential landscape associated with conductors whose cross sectional radius is comparable to the loop length^18^,^32^. In such a case, the induced current distributes itself unevenly across the conductor and produces a magnetic field that differs significantly from the one produced by a single filament, having direct impact on the quality of the trapping potential (see Supplementary Methods for details about calculation of the current distribution within metallic and superconducting loops^32^,^33^). An illustration of these effects is shown in Fig. 5, where we consider circular loops with square and circular cross sections made of two different conducting materials commonly used in atom-chip experiments: gold (Au) and superconducting niobium (Nb) (ref. 32).

In the case of a normal conductor, the combination of small skin depth at high frequency with a radially dependent magnetic flux pushes the induced current towards the outer edge of the conductor, spreading the current along its surface (see Supplementary Fig. 1). For superconducting loops, the Meissner effect, here described by the London equations^33^, contributes to a more marked confinement of the current close to the conductor surface (see Supplementary Fig. 2). In both cases, as a consequence of distributing the current over a wide area, the gradient of the magnetic field is reduced in comparison with the single-filament case. In terms of the atomic potential landscape, this translates to modifying the trapping position (that is, the centre of the quadrupole field distribution) and reducing its tightness (here quantified through the trap frequency along the *x* direction, *ν*_*x*_). Our numerical results indicate that both position and trap frequency, although dependent on the conducting material and cross sectional shape, do not vary strongly with these parameters. In both cases, the most relevant parameter is the thickness of the conductor, favouring the use of thin conductors to produce strong trapping potentials.

### Experimental considerations

The design of atom-chips including current carrying elements is limited by several technical issues that restrict the range of experimentally accessible parameters^5^. In the present case, for example, the goal of obtaining the tightest possible trap, via small detuning or large driving fields, should be balanced against an increase in heating of the conductors and atom-loss rates. In what follows, we briefly consider these two problems.

Ohmic loses due to the induced current must be restricted to avoid thermal destruction of the conductive loop, or undesirable alteration of the trapping track due to thermal deformations of the conductor. For typical experimental parameters, such as those in Fig. 3, the average current densities (see Fig. 5b and Supplementary Figs 1,2) are significantly lower than the maximal tolerable values demonstrated in experiments with normal and superconducting materials operating under DC and high-frequency conditions (≈10^6^ A cm^−2^)^34^,^35^, suggesting that the heat generated in our proposed trapping set-up can be efficiently transferred to the supportive structures of the device. Also, although our numerical results for heating power favours using thick conductors, this should be balanced against the higher trapping frequency and better thermal coupling achievable with thin wires, which can support large current densities and are also convenient for fabrication^5^.

We estimate non-adiabatic atom losses in our trapping set-up by considering an atom moving at speed *u* in the plane defined by the conducting loop. After the rotating-wave approximation, the atom-field interaction is described by the two-level Hamiltonian^8^:


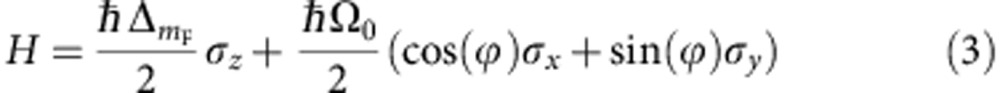


where *σ*_*i*_ with *i*=*x*, *y*, *z* are Pauli matrices, and the spatially dependent phase *ϕ* and Rabi frequency Ω_0_, are defined by the combination of the applied and induced fields. Atom-loss processes are modelled as transitions between the position-dependent eigenvectors of Hamiltonian equation (3), denoted by {|1›,|2›} in the present treatment^4^. Such dressed states consist of linear combinations of hyperfine states with the same projection of angular momentum *m*_F_ that depends on the amplitude of the magnetic field. For example, at the centre of the quadrupole field distribution, where the field is null, the dressed states |1›,|2› coincide with the hyperfine states |*F*, *m*_F_›,|*F*−1, *m*_F_›, while very far from the zero they are equal superposition of these two states. In the trapping geometry produced by a circular loop of inductance *L* and radius *a*, the rate of transitions between pairs of dressed states is approximately^4^:


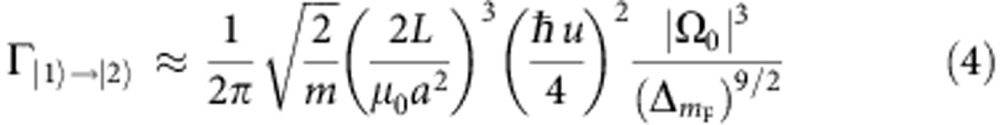


Under typical experimental conditions, for example, an atom moving with speed *u*≈10 mm s^−1^ (corresponding to a temperature of 1 μK), and for the trap configuration presented in Fig. 3, equation (4) predicts non-adiabatic transitions with a rate of ~10^−5^ Hz, allowing enough time for manipulation of the trapped atoms.

Feeding the external field into the conducting loop presents another potential challenge. However, in the case of ^6^Li and light atoms, the driving frequency falls in the MHz range, where several well-known techniques can easily be employed^5^. For the case of Rb and Cs isotopes, the driving field should have a frequency in the GHz range, for which near-surface fields of a microwave source could be suitable^30^,^34^,^35^,^36^. For example, a sufficiently uniform field of 2 G at a frequency of 6.8 GHz can be generated by a feeding structure consisting of a single-turn circular conductor of radius 1.7 mm, positioned concentrically on the same chip as the inductive loop. The dimensions of this feeding conductor are an order of magnitude larger than the inductive loop, and it is sufficiently far away that a small break for connecting a coaxial signal will not cause significant end effects near the smaller loop. Moreover, the amplitude of the produced field is uniform over the smaller loop at the <1% level (see Supplementary Fig. 3). The inductance of the feeding loop is ~6 nH, with a resistance ~0.2 Ω, due to the 1 μm skin depth at this frequency. The inductive impedance of the feeding loop (~250 Ω) can be cancelled at resonant microwave frequencies by a small capacitor of ~0.1 pF. In addition, a resistor in series with the feeding loop provides control of the resonance full-width-half-maximum, which naturally is ~7 MHz. The feeding loop needs to carry a current of ~0.5 A to generate a 2 G field at its geometrical centre, which is achievable using a transformer to drive the low impedance load via a (high impedance) commercial microwave source and amplifier^34^,^35^.

### Possibilities for loading the induced trapping loop

**Figure 6:**
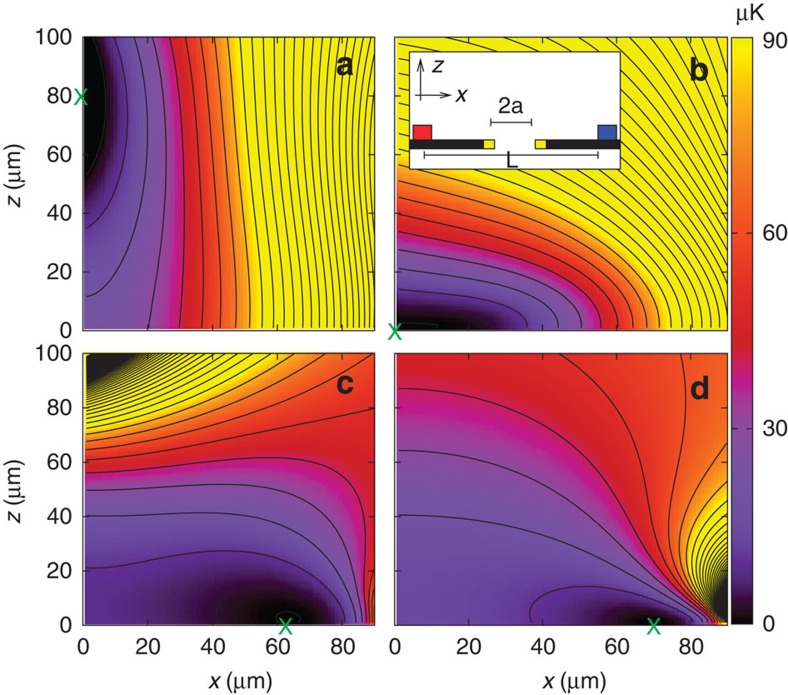
Loading scheme. Contours of the potential energy resulting from a two-wire magnetic trap plus an induced ring trap. The inset in panel (**b**) shows a cross section of the configuration for loading of the ring trap. It consists of two parallel wires carrying DC currents in opposite directions (blue and red squares) separated by a distance *L*=400 μm and a central ring of radius *a*=100 μm (yellow squares). For each panel, the parameters have been adjusted to produce a trap at different locations, indicated by a green cross. (**a**) *B*_DC,*z*_=20 G, *B*_DC,*y*_=0.5 G, *B*_AC_=0.0, *I*_left_=*I*_right_=1.3 A, setting the trap at **r**=(0, 80) μm (**b**) *B*_DC,*z*_=20 G, *B*_DC,*y*_=0.5 G, *B*_AC_=0.0, *I*_left_=*I*_right_=1.0 A, with the trap located at **r**=(0, 0). (**c**) *B*_DC,*z*_=1 G, *B*_DC,*y*_=0.5 G, *B*_AC_=2.0 G, *I*_left_=*I*_right_=0.1 A, Δ_0_/2*π*=0.6 MHz, with **r**=(63, 0) μm (**d**) *B*_DC,*z*_=1 G, *B*_DC,*y*_=0.0 G, *B*_AC_=2.0 G, *I*_left_=*I*_right_=0.0 A, Δ_0_/2*π*=0.66 MHz, with **r**=(68, 0) μm. Contours are separated by 10 μK, starting at 1 μK.

Atoms can be transferred to the induced trapping loop using an auxiliary near-surface magnetic trap, as commonly done in other micro-trapping geometries^5^. Following a slow variation of the trapping parameters, the potential landscape is deformed and atoms can be transported between regions separated by several hundreds of microns. For example, a typical magnetic microtrap, created by a set of conductors carrying DC currents and a uniform static field, can initially hold the atoms above the centre of the trapping loop. Subsequent modification of the current and applied fields deforms the trap and places the atoms in a region of significant influence of the induced ring trap. This procedure is schematically shown in Fig. 6, where we plot contours of the potential energy produced by the combination of a two-wire magnetic trap (see figure caption) with the energy shift caused by the AC field distribution. The figure shows four snapshots of a sequence that transport atoms from a position above the centre of the ring to the plane of the centre of the induced trapping region. The sequence starts with a magnetic trap centred above the ring, (**r**_0_=(0,0,*z*_0_)) (panel a), then, by adjusting the currents, the amplitude of applied fields and the driving frequency, the trap centre moves towards the plane of the ring, (**r**_1_=(0,0,0)) (panel b). The loading scheme then proceeds to slowly increase the field producing the induced ring trap and simultaneously reduce the strength of the magnetic trap (panels c and d). To reduce atom losses during the loading process, all parameters defining the trapping potential should be selected in such a way that the trap depth and frequencies are approximately constant through the procedure. A similar sequence can be planned by replacing the initial magnetic trap with a mirror-MOT (as in ref. 37), having the advantage of providing a uniform distribution of atoms along the trapping loop.

## Discussion

In summary, we show that complex one-dimensional guides for ultracold matter can be defined by inductive effects over metallic and superconducting loops. A very flexible wave-guide shape is possible as the guide simply follows the curves of a metal track laid down as a loop on the surface of an atom-chip or carved into it. For operation, the loop should receive a magnetic field that oscillates near to resonance with the hyperfine splitting of the atomic ground state of the atoms. The field induces an electric current on the conducting track without the need of leading wires that might introduce undesired asymmetries in the potential landscape. The combined applied and induced fields form a trapping structure for the atoms.

Our numerical investigations indicate that the experimental realization of this type of trap is realistic with current technology, predicting trapping frequencies varying from a few hundred Hz to a few kHz. This allows applications such as exploring low-dimensional looped traps for Bose-Einstein condensates, novel quantum interference devices and multiply looped structures for cold atom gyroscopes. Interestingly, our scheme can produce overlapping trapping regions for two different hyperfine states, which might be of practical interest for experiments with atomic species where a low magnetic field Feshbach resonance is available, such as in ^6^Li.

## Methods

### Current distribution in circular conductors

We evaluate the current distribution in conducting loops with circular and square cross sections. The trapping configuration works in the regime of long-wavelength compared with the size of the loop, which allows us to use quasi-static Maxwell equations coupled to constitutive relations between the fields and the current (for example, Ohm’s law and London equations)^31^. We use the open-source package FEMM^38^, which implements a Finite-Element algorithm for magnetic problems, to calculate the current distribution in metallic conductors. The corresponding total field distribution is then employed to evaluate the trapping frequency as a function of the conductor width in Fig. 5.

We also consider superconducting loops of Niobium and calculate its current distribution, **J**(**r**). To do so, we adapted the procedure detailed in ref. 39 as follows: First, as the frequency of the applied magnetic field is much smaller than the superconducting gap, the current can be described by the London equation^33^:


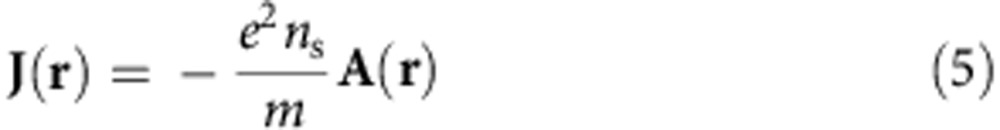


where *m* and *e* are the electron mass and charge, respectively, *n*_s_ is the density of superconducting electrons and the vector potential **A** satisfies the Coulomb gauge ∇·**A**=0. Second, the solution to quasi-static Maxwell equations for the magnetic field provides us a second relation between the vector potential and the current distribution:


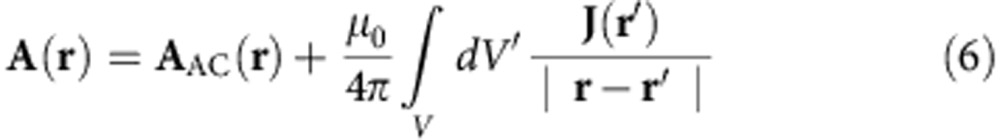


where **A**_AC_ is the vector potential corresponding to the applied field and the integral is limited to the volume where the current is defined^39^.

Then, combining these two equations and considering circular loops with homogeneous cross section, we obtain a relation between the applied flux of magnetic field across sections of loops with radius *ρ* and the current distribution:


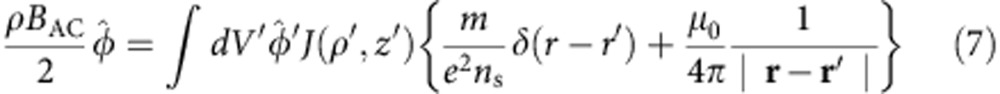


in which we have used cylindrical coordinates. In equation (7) *B*_AC_ is the amplitude of the oscillating applied field, *J*(*ρ*′, *z*′) is the current density at points (*ρ*′, *z*′) within the superconductor, 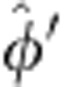 denotes a unitary vector along the azimuthal direction and the integral is restricted to the volume of the superconducting loop. After discretizing the superconductor cross section, we obtain an algebraic problem relating the currents passing through finite cross sections of the conductor and the applied flux. Finally, this set of equations is solved with a standard computing package for linear algebra (LAPACK). A fully detailed description of this procedure is shown in Supplementary Methods.

## Author contributions

B.M.G. and A.S.A. conceived the trapping scheme discussed. G.A.S.-L. and K.A.B. performed theoretical and numerical analysis. G.A.S.-L. and B.M.G. wrote the manuscript. All authors discussed the results and implications, and commented on the manuscript at all stages.

## Additional information

**How to cite this article:** Sinuco-Leon, G. A. *et al*. Inductively guided circuits for ultracold dressed atoms. *Nat. Commun.* 5:5289 doi: 10.1038/ncomms6289 (2014).

## Supplementary Material

Supplementary Information Supplementary Figures 1-4, Supplementary Methods and Supplementary References
