# Supplementary material for: Inductively guided circuits for ultracold dressed atoms
Source: Nat Commun. 2014 Oct 28;5:5289. doi: 10.1038/ncomms6289 (PMC4220492; doi:10.1038/ncomms6289)
Supplement: Supplementary Information — Supplementary Figures 1-4, Supplementary Methods and Supplementary References [file ncomms6289-s1.pdf]

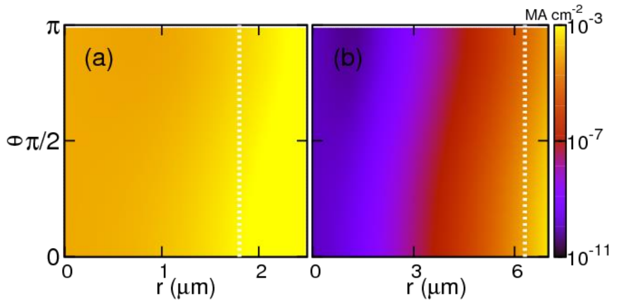

Supplementary Figure 1. Distribution of induced current in a normal metal. Current density distribution across the cross-section of a ring of gold with thickness (**a**) 2.5 μm and (**b**) 7 μm. In both cases, the ring radius is a = 100 μm, B_{AC} = 2 G and $\omega = 2\pi \times 6.8$ GHz. The vertical dashed line indicates the position of the skin-depth for the angular frequency ω. The axes correspond to the system of polar-coordinate defined with origin at the centre of the conductor cross-section.

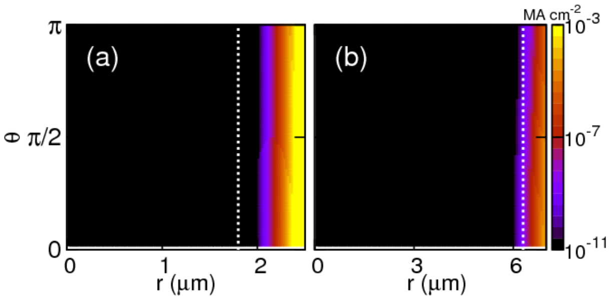

Supplementary Figure 2. Distribution of current in superconducting Nb. Current density across the cross-section of superconducting loops of Niobium with thickness (**a**) 2.5 μm and (**b**) 7 μm. In both cases, the ring radius is a = 100 μm, B_{AC} = 2G and $\omega = 2\pi \times 6.8$ GHz. The vertical dashed line indicates the position of the skin-depth of gold for the angular frequency ω. The axes corresponds to the system of polar-coordinates defined with origin at the centre of the conductor cross-section.

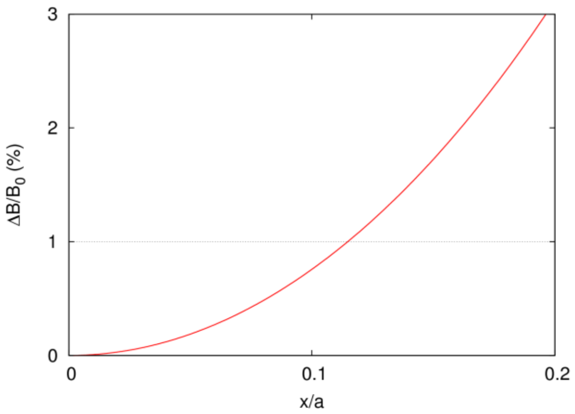

Supplementary Figure 3. Variation of the magnetic field produced by circular current. Variation of the magnetic field produced by a circular current flow (single filament) of radius a, relative to the field at the loop centre, B_0, and along the plane of the loop.

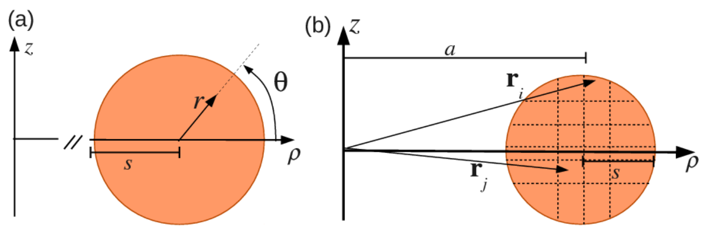

Supplementary Figure 4. Coordinate system for current distribution. (**a**) The current density in circular rings are evaluated at points defined through the polar coordinate system with origin at the centre of the conductor cross-section. (**b**) Cylindrical coordinates used to define the Maxwell and ring constitute equations. In this work, we consider conductors with rotational symmetry around the z axis. In both panels, circular regions represent the conductor cross-section, and the external field oscillates along the z axis.

SUPPLEMENTARY METHODS

DISTRIBUTION OF INDUCED CURRENTS IN CONDUCTORS OF FINITE CROSS-SECTION

We present details of a numerical procedure to determine the distribution of induced current within conducting rings with uniform cross-section. We focus on the case of magnetic fields oscillating at low frequencies such that the associated wavelength ($\lambda = c/\nu$) is much larger than the size of the loop, and apply a quasi-static approximation to the Maxwell equations for the electromagnetic field [1, 2]. First, we present results for the current distribution in metallic rings, taking parameters corresponding to gold, obtained using the open-source software FEMM. Second, we detail a procedure to evaluate the current distribution in superconducting rings in the limit of the London equation. In this last case, we consider parameters corresponding to superconducting Niobium and adapt the methods in references [3] and [4] to the present problem.

The current distribution is evaluated using the coordinate systems in Supplementary Fig. 4. Exploiting the circular symmetry of the ring, the current density is calculated at points (r,θ), defined by a polar-coordinate system with origin at the ring cross-section centre, as shown in Supplementary Fig. 4(a). The Maxwell equations associated to time-dependent magnetic fields and its sources are coupled to a constitutive relation between the fields and the current in the ring. In our case, such constitutive equations correspond to the Ohm law for metallic conductors, and the London equation for superconducting materials. The resulting set of coupled equations are conveniently expressed in the cylindrical coordinate system with origin at the centre of the ring, as shown in Supplementary Fig. 4(b).

Metallic rings

A time-variation of magnetic flux across a metallic ring induces an electric current whose distribution depends on the properties and geometry of the ring and the frequency of the field. For a harmonic variation of the magnetic field with angular frequency ω, the quasi-static Maxwell

equation for the vector potential is:

$$\nabla \times \nabla \times \mathbf{A}(\mathbf{r}) = -i\sigma\omega \mathbf{A}(\mathbf{r}) \quad (1)$$

where σ is the ring conductivity [2].

We use the open-source software package FEMM [5] to solve Eq. (1), for gold rings of various sizes. We setup an external magnetic field of amplitude B_{AC} = 2 G, oscillating at a frequency $\omega = 2\pi \times 6.8$ GHz along the z direction. Supplementary Fig. 1 shows the distribution of induced current for rings of thickness s = 2.5 μm and s = 7 μm, (a = 100 μm). In rings of thickness comparable to the skin-depth at high frequencies (i.e. of the order of 1 μm), the current distributes almost homogeneously across the whole area of the cross-section. In the case of thicker rings, the current concentrates along the conductor surface leaving the conductor centre free from current flow. This confinement of the current causes the power dissipated by the electric flow to diminish with the thickness of the conductor (see main text – figure 5), due to the decrease of the effective area where the current flow occurs.

FEMM also provides the magnetic field distribution, which we use to evaluate the trapping frequencies shown in the main text.

Superconducting rings.

We consider superconducting rings of uniform cross-section, described by the London theory [1], where the supercurrent and the potential vector are related by:

$$\mathbf{J}(\mathbf{r}) = -\frac{e^2 n_s}{m}\mathbf{A}(\mathbf{r}) \quad (2)$$

with m and e the electron mass and charge, respectively, and n_s the density of superconducting electrons. By using this expression, we neglect non-local effects on the current distribution and restrict our calculations to field frequencies smaller than the superconducting gap (typically of the order of a few ~ 100GHz) [1].

Under quasi-static conditions, the total vector potential corresponding to an external field and a current distribution is:

$$\mathbf{A}(\mathbf{r}) = \mathbf{A}_{\mathbf{AC}}(\mathbf{r}) + \frac{\mu_0}{4\pi}\int dV' \frac{\mathbf{J}(\mathbf{r}')}{|\mathbf{r}-\mathbf{r}'|}, \tag{3}$$

where the integral is taken over the volume of the current-carrying conductors. $\mathbf{A}_{\mathbf{AC}}$ is the vector potential associated with the applied field which, in the case of a uniform magnetic field along the z axis is $\mathbf{A}_{\mathbf{AC}} = \frac{\rho B_{\mathbf{AC}}}{2}\hat{\boldsymbol{\varphi}}$, after imposing the Coulomb gauge condition $\nabla \cdot \mathbf{A}_{\mathbf{AC}} = 0$ [1].

Superconducting rings with homogeneous cross-section have a current distribution independent of the azimuthal angle φ, and flows tangentially to the perimeter of the conductor along the direction defined by $\hat{\boldsymbol{\varphi}}$. This argument and Eq. (2) allow us to write Eq. (3) in the form:

$$\frac{\rho B_{\mathbf{AC}}}{2}\hat{\boldsymbol{\varphi}} = \int dV' \hat{\boldsymbol{\varphi}}' J(\rho', z') \left\{ \frac{m}{e^2 n_s}\delta(\mathbf{r}-\mathbf{r}') + \frac{\mu_0}{4\pi}\frac{1}{|\mathbf{r}-\mathbf{r}'|} \right\} \tag{4}$$

where we have used an elementary property of the Dirac delta distribution [4]. It is convenient to separate the integral over the volume of the conductor into an integral over the conductor cross-section and one over its circumference (see Fig. 1(b)):

$$\int dV' \hat{\boldsymbol{\varphi}}' = \int\int d\rho' dz' d\mathbf{l}' \tag{5}$$

where $d\mathbf{l}' = \rho' d\varphi' \hat{\boldsymbol{\varphi}}'$. Thus Eq. (4) becomes:

$$\frac{\rho B_{\mathbf{AC}}}{2}\hat{\boldsymbol{\varphi}} = \int d\rho' dz' J(\rho', z') \int Q(\mathbf{r}-\mathbf{r}') d\mathbf{l}' \tag{6}$$

with $Q(\mathbf{r},\mathbf{r}')$ defined as:

$$Q(\mathbf{r},\mathbf{r}') = \frac{m}{e^2 n_s}\delta(r-r') + \frac{\mu_0}{4\pi}\frac{\mathbf{1}}{|\mathbf{r}-\mathbf{r}'|} \tag{7}$$

Equation (4) can be recast in terms of magnetic flux across the loop C defined by $\{\mathbf{r} = (\rho,\varphi,z) \text{ with } \varphi \in [0,2\pi)\}$, using the relation $\Phi_C = \int_C \mathbf{A}\cdot d\mathbf{l}$:

$$\pi\rho^2 B_{\mathrm{AC}} = \int d\rho' dz' J(\rho', z') \int_C \int_{Ring} Q(\mathbf{r},\mathbf{r}') d\mathbf{l}' \cdot d\mathbf{l} \tag{8}$$

This equation implies that the magnetic flux across the loop C, created by the current distribution, compensates exactly the magnetic flux imposed by the external field. This corresponds to the well-known Meissner effect in superconductors, and implies that the induced

current adjusts instantaneously to cancel the total flux of magnetic field across any loop defined within the superconducting ring.

To obtain a solution of Eq. (8), we discretize the conductor cross-section in elements of area ΔA_i centred at positions $\mathbf{r}_i$, as schematically shown in Supplementary Methods Fig. 1(b). In discrete from, Eq. (8) becomes:

$$\pi\rho_i^2 B_{\mathbf{AC}} = \sum L_{i,j} I_j \tag{9}$$

where $I_j = J_j \Delta A_j$ is the current flowing across the j-th are element ΔA_j, and:

$$L_{i,j} = \int\int Q(\mathbf{r}_i, \mathbf{r}_j) d\mathbf{l}_i \cdot d\mathbf{l}_j \tag{10}$$

is the mutual inductance between the i-th and j-th loops, which for $i \neq j$ becomes:

$$L_{i,j} = \frac{\mu_0 \rho_i \rho_j}{4\pi} \int du \frac{\cos u}{\left(\rho_i^2 + \rho_j^2 + (z_i - z_j)^2 - 2\rho_i 2\rho_j \cos u\right)^{1/2}} \tag{11}$$

This integral is evaluated following [4].

Following [3], the self-inductance $L_{i,i}$ can be approximated by:

$$L_{i,i} = \mu_0 \rho_i \left[\log\left(\frac{8\rho_i}{R}\right) - \frac{7}{4}\right] + \mu_0 \lambda^2 \frac{2\pi\rho_i}{\Delta A_i} \tag{12}$$

which includes the kinetic inductance term with $\lambda^2 = \dfrac{m}{\mu_0 n_s e^2}$.

We consider a superconducting ring of size a = 100 μm, and circular cross-section in the range 1-20 μm. For Niobium, the London penetration depth is $\lambda \approx 100$ nm [3]. Supplementary Fig. 2 presents the current distribution in rings with s = 2.5 μm and s = 7 μm (a = 100 μm), for an applied field B_{AC} = 2 G. In comparison to the case of metallic conductors shown in Supplementary Fig. 1, the current distribution concentrates more strongly near the surface of the conductor. Nevertheless, the impact on the trapping properties of the ring trap discussed in the main text is similar in both cases (see main text - Figure 5).

Supplementary References

[1] M. Tinkham, Introduction to superconductivity, Vol. 89 (McGraw-Hill New York, 1975).

[2] J. Jackson, Classical electrodynamics, 3rd ed. (John Wiley & Sons, 1998).

[3] D. Cano, B. Kasch, H. Hattermann, R. Kleiner, C. Zimmermann, D. Koelle, and J. Fortagh, Phys. Rev. Lett., 101, 183006 (2008).

[4] E. H. Brandt and J. R. Clem, Phys. Rev. B, 69, 184509 (2004).

[5] D. C. Meeker, Finite Element Method Magnetics, edited by V. D. Build, (2006).
